# FOXP2, retinoic acid, and language: a promising direction

**DOI:** 10.3389/fncel.2014.00387

**Published:** 2014-11-13

**Authors:** Antonio Benítez-Burraco, Cedric Boeckx

**Affiliations:** ^1^Department of Spanish Philology and its Didactics, University of HuelvaHuelva, Spain; ^2^Catalan Institute for Advanced Studies and Research (ICREA)Barcelona, Spain; ^3^Department of Linguistics, Universitat de BarcelonaBarcelona, Spain

**Keywords:** language, neurite outgrowth, FoxP2, retinoic acid, neuron differentiation

Devanna et al. (2014) have demonstrated that FOXP2 mimics, and actually potentiates, retinoic acid (RA) induction of genes involved in neural differentiation. At the physiological level this effect results in an increase of neurite outgrowth and branching, and in a reduction of neuronal migration. The authors highlight the importance of RA signaling for brain growth and differentiation, and the relevance of *FOXP2* for language. Specifically, the authors' interest focuses on the upregulation of *RARβ* by FOXP2 in the striatum, where the primary pathology is located in people bearing a defective copy of *FOXP2*, known to give rise to language disorders (see Graham and Fisher, 2013 for review). Devanna et al.'s study adds to the literature showing that RA plays an important role in brain plasticity (Luo et al., 2009), learning and memory (Etchamendy et al., 2003; Jiang et al., 2012), and we find this research direction promising. In our opinion the link between RA, *FOXP2*, and language could be made more robust by taking advantage of information already available in the literature, which we wish to highlight here. In doing so, we hope to encourage further experimental testing in this area.

Recently we have assembled a set of genes that we predict to be implicated in the refinement of the connectivity between sub-cortical and cortical structures, as well as the interface between brain growth and skull formation, and which may underlie our species-specific “language readiness” (Boeckx and Benitez-Burraco, 2014). Interestingly, in the context of Devanna et al.'s study, several of the genes belonging to our list are related to the RA signaling pathway, to *FOXP2*, or to both them. These links, if further explored and eventually mapped onto particular aspects of neural function and brain development could reinforce Devanna et al.'s findings and help us better understand the molecular underpinnings of human language.

Our set of genes is centered on *RUNX2*, which controls different aspects of skull and brain development (Stein et al., 2004; Reale et al., 2013) and whose promoter region shows two derived alleles in modern humans (Perdomo-Sabogal et al., 2014). One of the RUNX2 targets is *CRABPII* (Wu et al., 2014), a RA signaling component highlighted by Devanna et al. Another target of RUNX2, and also a gene regulated by RA, is *HES1* (Suh et al., 2008). The HES1 pathway is related to craniofacial development (Wen et al., 2013), the differentiation of GABAergic neurons, standardly regarded as critical for the maintenance of our species-specific cognitive profile (Long et al., 2013), and the development of dopaminergic neurons, routinely mentioned in the literature on motor behavior and vocal learning (Kameda et al., 2011). Moreover, *HES1* is transcriptionally regulated by the SLIT/ROBO pathway (Borrell et al., 2012), which is impaired in language disorders and autism (Suda et al., 2011; St Pourcain et al., 2014; Tran et al., 2014) and which is implicated in the establishment of the vocal learning neural circuits in birds (Wang, 2011). Importantly, the SLIT/ROBO pathway interacts with FOXP2: both Vernes et al. (2007) and Konopka et al. (2009) have identified *SLIT1* as a direct downstream target of FOXP2. Finally, among the RUNX2 targets identified by Kuhlwilm et al. (2013), two genes (*NLGN1* and *ITPR1*) are both candidates for autism spectrum disorder and targets of RORA1, a major isoform of the RA-related orphan receptor-alpha (RORA) protein in the human brain, and also a candidate for autism (Sarachana and Hu, 2013). Interestingly, among the genes highlighted by Sarachana and Hu (2013) one also finds candidates for language disorders, like *CYP19A1* (Anthoni et al., 2012), and several targets of FOXP2, like *NTRK* and *A2BP1* (Konopka et al., 2009). The latter gene is also a target of the neural splicing factor FOX-1, related to many neurodevelopmental diseases and one of the FOXP2 targets that show strong signals of selection in modern humans (Ayub et al., 2013).

Finally, another gene also highlighted by Devanna et al. is *ASCL1*, known to be involved in RA signaling. According to the authors, both FOXP2 and RA strongly downregulate *ASCL1*. We have found that ASCL1 regulates the DLX suite and the development of most neocortical GABAergic neurons (Letinic et al., 2002). We argued in Boeckx and Benitez-Burraco (2014) that *DLX1* and *DLX2* are likely to play an important role in the formation of a language-ready brain. Interestingly, *Ascl1*, *Dlx1*, *Dlx2*, and Foxp2's target *Nkx2-1* regulate the development of the basal ganglia in mice (Anderson et al., 1997; Casarosa et al., 1999). Moreover, one partner of ASCL1 is DLL1 (Nelson and Reh, 2008), linked to many of the genes involved in vocal learning (Wang, 2011). It is worth noting in this context that Devanna et al. have found that both RA and FOXP2 dowregulate *DLL3*. Although data for *DLL1* are not available, we observe here that in mice mutant for *Ascl1* (lacking discrete neuronal populations of the cerebral cortex and the basal ganglia) neither *Dll1* nor *Dll3* are expressed in the ventral telencephalon (Casarosa et al., 1999). Lastly, we wish also highlight that ASCL1 interacts with POU3F2, a protein that regulates the upper-layer neuronal migration and identity during the development of the neocortex (McEvilly et al., 2002). *POU3F2* has been linked to developmental and language delays, intellectual disability, schizophrenia and autism spectrum disorders (Lin et al., 2011). It has been shown that modern humans exhibit a (nearly fixed) substitution in intron 8 of *FOXP2* that affects a binding site for POU3F2, which results in a less efficient way of activating transcription of *FOXP2* (Maricic et al., 2013). POU3F2 also interacts with PQBP1 (Waragai et al., 1999), a protein involved in neurite growth and neuron projection, and linked to intellectual disability (Wang et al., 2013). (As we noted at the outset, Devanna et al. highlight that FOXP2 promotes increased neurite outgrowth and impair neuronal cell migration in response to RA.)

In sum, we regard the findings by Devanna et al. of outstanding interest concerning the genetic, molecular, and physiological underpinnings of language. We believe that these findings could be reinforced if the links with the genes mentioned in this commentary are explored and confirmed regarding specifically the development and function of brain areas involved in language processing, and we hope that this commentary will encourage geneticists to do so.

## Conflict of interest statement

The authors declare that the research was conducted in the absence of any commercial or financial relationships that could be construed as a potential conflict of interest.
